# Urinary Sediment mRNA Level of CREBBP and CYBA in Children With Steroid-Resistant Nephrotic Syndrome

**DOI:** 10.3389/fimmu.2021.801313

**Published:** 2022-01-31

**Authors:** Wei Li, Xinyi Shou, Wenqing Xiang, Lin He, Lin Li, Haidong Fu, Jianhua Mao

**Affiliations:** ^1^ Department of Clinical Laboratory, The Children’s Hospital, Zhejiang University School of Medicine, National Clinical Research Center for Child Health, Hangzhou, China; ^2^ Department of Nephrology, The Children’ s Hospital, Zhejiang University School of Medicine, National Clinical Research Center For Child Health, Hangzhou, China

**Keywords:** mRNA, idiopathic nephrotic syndrome, steroid resistance, urinary sediment, CREBBP, CYBA

## Abstract

**Background:**

This study aimed to evaluate gene expression patterns in urinary sediment samples of children with steroid-resistant nephrotic syndrome (SRNS).

**Methods:**

The messenger RNA (mRNA) levels of 770 immune-related genes were detected using a NanoString nCounter platform. To verify the NanoString results, quantitative analysis of nine gene mRNAs was performed using real-time RT-PCR in more samples.

**Results:**

Firstly, compared with the steroid-sensitive nephrotic syndrome (SSNS) group (*n*=3), significant changes were observed in the mRNA level of 70 genes, including MAP3K14, CYBA, SLC3A2, CREB-binding protein (CREBBP), CD68, forkhead box P1 (FOXP1), CD74, ITGB2, IFI30, and so forth, in the SRNS group (n=3). A total of 129 children with idiopathic nephrotic syndrome (INS), 15 with acute glomerulonephritis, and 6 with immunoglobulin A nephropathy (IgAN) were enrolled to verify the NanoString results. Compared with patients with IgAN, those with INS had significantly lower levels of FOXP1 (P=0.047) and higher levels of CREBBP (P=0.023). Among SSNS, the mRNA level of ITGB2 was significantly lower in the non-relapse group than in the non-frequent relapse and frequent-relapse groups (P=0.006). Compared with the SSNS group, CREBBP was significantly elevated in the SRNS group (P=0.02). Further, CYBA significantly decreased in the SRNS group (P=0.01). The area under the curve (AUC) for CREBBP and CYBA was 0.655 and 0.669, respectively. CREBBP had a sensitivity of 83.3% and a specificity of 49.4% and CYBA had a sensitivity of 58.3% and a specificity of 83.1% to rule out SSNS and SRNS. The diagnosis value was better for CREBBP+CYBA than for CREBBP or CYBA alone, indicating that the combination of CREBBP and CYBA was a more effective biomarker in predicting steroid resistance (AUC=0.666; sensitivity=63.9%; specificity=76.4%).

**Conclusions:**

This study was novel in investigating the urinary sediment mRNA level in children with INS using high-throughput NanoString nCounter technology, and 70 genes that may relate to SRNS were found. The results revealed that the urinary sediment mRNA level of ITGB2 was significantly lower in the non-relapse group than in the non-frequent relapse and frequent-relapse groups. Meanwhile, CREBBP was significantly elevated and CYBA was significantly lowered in the SRNS group compared with the SSNS group.

## Introduction

Idiopathic nephrotic syndrome (INS) is the most common glomerular disease among children. It is characterized by massive proteinuria, hypoalbuminemia, and edema (1). Childhood INS is most commonly caused by one of two idiopathic diseases: minimal change disease (MCD) and focal segmental glomerulosclerosis (FSGS) (2). A third distinct type, membranoproliferative glomerulonephritis (MPGN), is rare among children (3). More recently, MCD and FSGS are considered to fall within a spectrum of disease, with MCD representing an earlier stage that is more responsive to treatment and FSGS considered a more advanced and resistant stage of disease (4). The pathogenesis of INS is also unclear but related to autoimmune dysfunction, previous studies suggested that T cell dysfunction is one of the important mechanisms of MCD incidence (5, 6). Many studies also described that various alterations in cytokine production in process of INS, such as IL-4, IL-13, TNF-α (7–9).

The most commonly used drugs in treating INS are steroids (10). INS is clinically classified based on the response to corticosteroid therapy. Between 80% and 90% of children over the age of 1 year presenting with INS respond to treatment with steroids within 4 weeks [steroid-sensitive nephrotic syndrome (SSNS)], while the remaining 10%–20% are nonresponsive and classified as having steroid-resistant nephrotic syndrome (SRNS) (10). SRNS is more likely to be associated with FSGS pathology, and has more possibility of progressing to end-stage renal disease (ESRD). The probability of progression to ESRD was 8%–35% in 5 years after diagnosis and 24%–66% in 15 years after diagnosis (11, 12).

Urine and urinary sediment samples can be used as useful specimens to study INS, such as cells, microRNA (miRNA), and messenger RNA (mRNA) (13, 14). Besides the clinical routine test, urinary podocytes may be a useful diagnostic indicator for differentiation between MCD and FSGS (15). Urinary miR-30a-5p increases in children with INS (16), and the levels of urinary miR-29a, miR-192, and miR-200c have characteristic alterations among patients with different causes of INS (17). Previous studies suggested that the mRNA level of target genes in urinary sediment samples has been suggested as a noninvasive marker of renal damage. The levels of urinary nephrin and podocin mRNA were reduced in patients with MCD and probably FSGS, and the magnitude of reduction correlated with the degree of proteinuria (18). Urinary collagen type 1 alpha 1 mRNA level was elevated in patients with nephrosis irrespective of the pathological diagnosis, and it correlated with proteinuria and histological scarring, and inversely with renal function (19). High-throughput studies on the mRNA level of urinary sediment samples in children with INS are lacking. Therefore, in this study, NanoString nCounter gene expression was used for high-throughput detection of the mRNA level in the urinary sediment cells of children with NS.

## Materials and Methods

### Population

The diagnosis of INS was based on the presence of edema, 24-h urinary protein excretion of ≥50 mg/kg, morning urinary protein/creatinine of >2 mg, hypoalbuminemia of <25 g/L and the disease of unknown causing (20). All children with INS received the standard steroid therapy and were classified into two categories, SSNS and SRNS, on the basis of their clinical responses toward steroids. The SSNS group included patients with negative urinary protein for ≤4 weeks in those treated with sufficient prednisone [2 mg/(kg · d) or 60 mg/(m · d)]. The SRNS group included patients who failed to achieve remission after 4 weeks of daily sufficient prednisone (20). Further, the relapse group included patients in whom the quantity of urinary protein was ≥50 mg/kg, or the urinary protein/creatinine (mg/mg) of morning urine was ≥2.0, or the morning urinary protein changed from negative to (+)–(+ + + +) for three consecutive days. The non-relapse group included patients in whom INS no recurred within 1 year after the first complete remission. The non-frequent relapse group included patients in whom INS recurred once within 6 months or one to three times within 1 year after the first complete remission. The frequent relapse group included patients in whom INS recurred two times or more within half a year, or four times or more within 1 year in the course of the disease (20). Immunoglobulin A nephropathy(IgAN) and acute glomerulonephritis were used as other kidney diseases in this study. MCD, MPGN, and FSGS were confirmed by the pathological examination. The design of this prospective study and the methods used were approved by the ethics committee of the children’s hospital of Zhejiang university, school of medicine (2020-IRB-057).

### RNA Extraction

From January 2019 to August 2020, the urine sample was collected from each patient (INS, IgAN and acute glomerulonephritis) before steroid treatment. First, 10 mL of urine samples were centrifuged at 3000g for 10 min at 4°C immediately after collection. The supernatant was discarded, and the urinary cell pellet mixture were mixed with RNAlater (Invitrogen, CA, USA) and stored at −80°C until further use. RNA was extracted from the cell mixture using an EX3600 instrument with the magnetic bead method (catalog: Z-ME-0044, Shanghai Zhijiang Biotechnology Ltd. Co).

### NanoString Analysis

Gene expression analysis was conducted on a NanoString nCounter gene expression platform (NanoString Technologies, WA, USA) (21). A custom code set consisting of a 770-gene panel related to disease, chronic inflammatory disease, immune-related adverse events (comprehensive assessment of 35 pathways and processes associated with disease and chronic inflammatory disease) was used in this study. A final volume of 5 µL of total RNA (>20 ng) per sample was mixed with a 3′ biotinylated capture probe and a 5′ reporter probe tagged with a fluorescent barcode from the custom gene expression code set. Probes and target transcripts were hybridized overnight at 65°C for 12–16 h according to the manufacturer’s recommendations. Hybridized samples were run on the NanoString nCounter preparation station using a high-sensitivity protocol. Excess capture and reporter probes were removed, and transcript-specific ternary complexes were immobilized on a streptavidin-coated cartridge. The samples were scanned at maximum scan resolution on an nCounter Digital Analyzer.

Gene expression data for each individual sample were normalized by quantile normalization. Gene counts collected from the NanoString scanner were used as input variables with a reference distribution generated using a pool of counts from all samples and 770 genes (excluding data from positive and negative control probes). After performing quantile normalization, a log10 transformation was applied, and signature scores were calculated by averaging the included gene signatures. The log10 count of each gene on the platform was normalized by subtracting the arithmetic mean of log10 counts of the housekeeping genes (18 genes).

### Real-Time Quantitative Reverse Transcriptase–Polymerase Chain Reaction

An UltraSYBR One-Step quantitative reverse transcriptase–polymerase chain reaction (qRT-PCR) High ROX reagent (catalog: CW2624S, NYbio, China) was used to quantify the mRNA levels. Briefly, the quantification of the relative mRNA abundance was performed using an ABI StepOnePlus Detector System (Applied Biosystems, CA, USA). In brief, 2 µL of the total RNA was mixed with 200nM of primers and one-time SYBR Green Universal Master Mix in each reaction. The assay was according to the following protocol: 45°C for 10 min, 95°C for 5 min, followed by 40 cycles of 95°C for 10 s and 60°C for 45 s. Glyceraldehyde 3-phosphate dehydrogenase (GAPDH) was used as a housekeeping gene. The relative mRNA abundance in the patient group was calculated using the 2^−ΔΔCt^ method. The primers in this study were shown in **Table 1**.

**Table 1:** The primers used in this study.

Gene	Forward primer (5’ - 3’)	Reverse primer (5’ - 3’)
*MAP3K14*	TTCATGGAGCTGCTGGAAGG	GGAGCACGTTGTCAGCTTTG
*CYBA*	GCATCTACCTACTGGCGGC	TTGATGGTGCCTCCGATCT
*SLC3A2*	GGATGCTCTGGAGTTTTGGC	CCGCAATCAAGAGCCTGTCT
*CREBBP*	AAACCAAACAAACCATCCTGG	CATTGGATTATTTCCCAGGG
*CD68*	CAGCACAGTGGACATTCTCG	CACTGGGGCAGGAGAAACTTTG
*FOXP1*	GGCAGGCCATTCTCGAATCT	GACGCACTGCATTCTTCCAC
*CD74*	TGCACCTGCTCCAGAATGC	TACTTTCGGTGGAGCGTCAG
*ITGB2*	ATGAGAGCCGAGAGTGTGTG	TGTTCCACTGGGACTTGAGC
*IFI30*	GGTCACCGTCAATGGGAAAC	GCTTCTTGCCCTGGTACAAC
*GAPDH*	CCTGTTCGACAGTCAGCCG	CGACCAAATCCGTTGACTCC

### Statistical Analysis

All Nanostring nCounter platform data were analyzed and compared by the nSolver Analysis Software, such as QC, normalization, gene signatures, and pathway enrichment. P ≤ 0.05 was the statistically significant difference between group of SSNS and SRNS. We used Student’s t-test or Wilcoxon rank sum test to compare the means of age between two groups. Chi square test were used for the differences of sex. P > 0.05 was considered as no significant difference. The real time RT-PCR results of the two groups were compared by t-test or Wilcoxon rank sum test. The real time RT-PCR results of more than two groups were compared by analysis of variance or Kruskal Wallis anova test, the pairwise comparison among the three groups was used Bonferroni correction. P ≤ 0.05 was the statistically significant difference. ROC curve was used to determine the effective area, sensitivity and specificity of candidate indexes. SPSS 22.0 was used for statistical analysis.

## Results

### NanoString Assay

**Figure 1:**
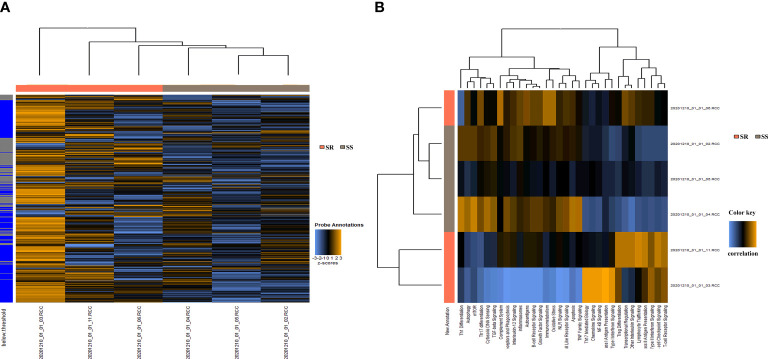
Gene signature development in urinary sediment samples from children with SSNS (*n* = 3) or SRNS (*n* = 3). **(A)** 770 Gene expression in each sample. **(B)** Important pathways between the SSNS and SRNS groups.

A set of baseline biopsies were performed in six patients with INS without steroid therapy enrolled in this study, including three with SSNS (including 2 boys and 1 girls, with a mean age of 5.2 ± 3.1 years) and three with SRNS (including 2 boys and 1 girls, with a mean age of 5.3 ± 2.8 years), to define immune-related gene expression signatures associated with response to steroids in children with INS. A one-sided *t* test was conducted to rank top genes associated with SSNS and SRNS, using a custom panel of 770 genes on a NanoString nCounter platform (NanoString Technologies Inc. WA, USA). As shown in **Figure 1A**, compared with the SSNS group, significant changes were observed in the mRNA level of 70 genes in the SR group, including MAP3K14, CYBA, SLC3A2, CREBBP, CD68, forkhead box P1 (FOXP1), CD74, ITGB2, IFI30, SMN1, and so forth. Positive genes were mapped into the pathway databases to confirm that these positive genes were involved in the biological processes between SSNS and SRNS. The results of pathway analysis indicated that the unigenes were related to 21 signaling pathways, particularly to T helper type 1 (Th1) differentiation, autophagy, mechanistic target of rapamycin (mTOR) and T-cell reporter signaling, T-cell checkpoint signaling, and type II interferon signaling pathways (**Figure 1B**).

### Real-Time RT-PCR

The top nine genes were chosen to confirm the expression of mRNAs using the NanoString nCounter assay. This study included 129 children with INS (including 96 boys and 33 girls, with a mean age of 5.6 ± 4.0 years, not including patients used by Nanostring nCounter), 15 children with acute glomerulonephritis (including 10 boys and 5 girls, with a mean age of 7.3 ± 3.9 years), and 6 children with IgAN (including 4 boys and 2 girls, with a mean age of 6.0 ± 1.2 years). Of the children with INS, 94 (72.1%) were classified as SSNS and 35 (27.9%) as SRNS. Among 90 children with SSNS, 30 (33.3%) were classified as non-relapse, 43 (47.8%) as non-frequent relapse, and 17 (18.9%) as frequent relapse. There is no significant difference in age and sex distribution among INS, IgAN and acute glomerulonephritis children.

**Figure 2:**
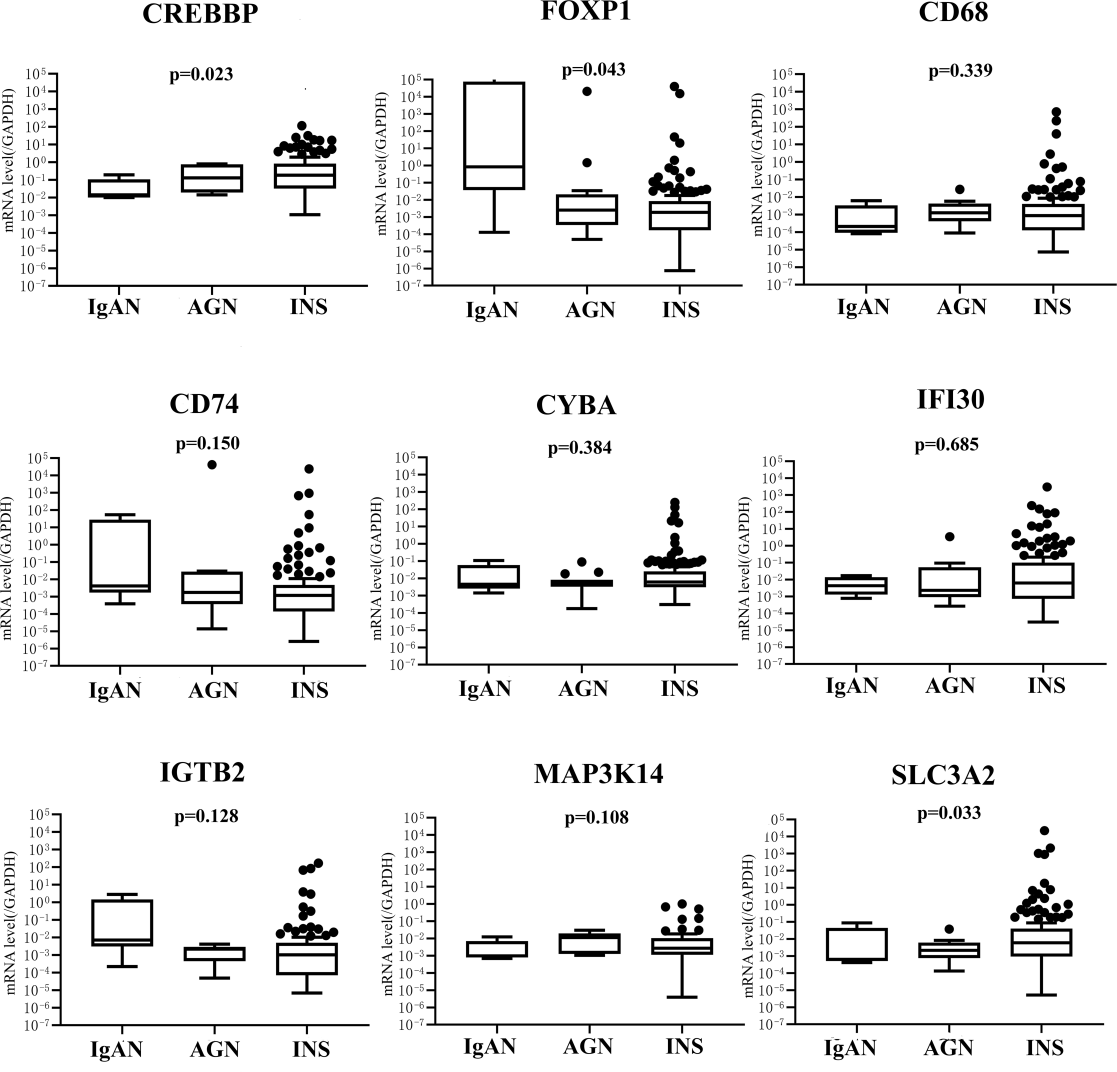
mRNA expression of nine genes in IgAN (*n* = 15), GN (*n* = 6), and INS (*n* = 129) groups. IgAN, IgA nephropathy; AGN, acute glomerulonephritis; INS, idiopathic nephrotic syndrome.

The mRNA levels of nine genes in the INS, acute glomerulonephritis, and IgAN groups are shown in **Figure 2**. Significant differences were observed in the expression levels of CREBBP, SLC3A2, and FOXP1 (*P* < 0.05). Compared with IgAN, patients with INS had significantly lower levels of FOXP1 (*P* = 0.047) and higher levels of CREBBP (*P* = 0.023).

**Figure 3:**
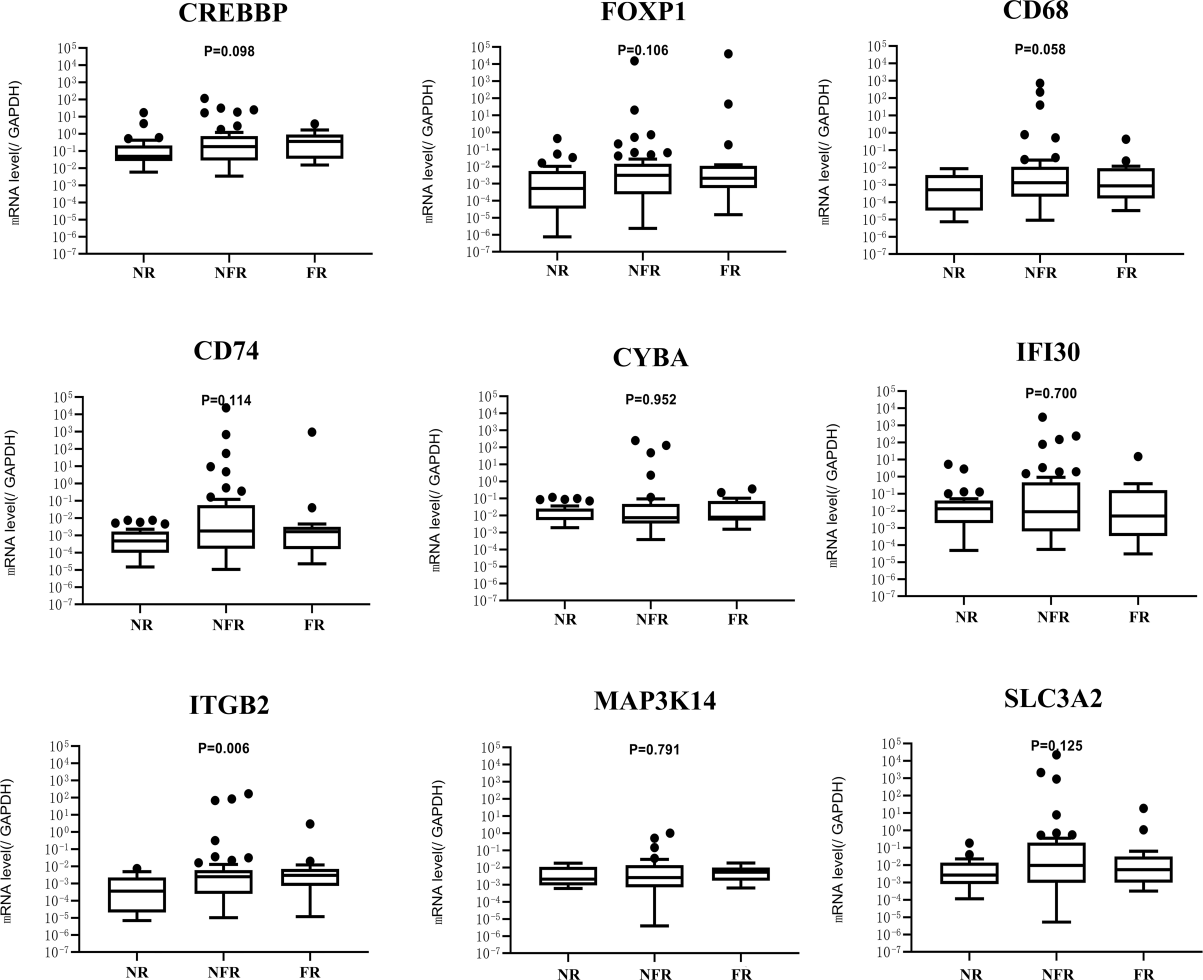
mRNA expression of nine genes in NR (*n* = 30),NFR (*n* = 43), and FR (*n* = 17) groups. NR, no relapse; NFR, non-frequent relapse; FR, frequent relapse.

According to pathological classification, the mRNA levels of nine genes were compared among the MCD (n=42), MPGN (n=5), and FSGS (n=3) groups. No significant difference was found in the distribution of genotypes among the three groups (*P* > 0.05, data not shown in this study). For children with INS, the mRNA levels of nine genes were compared in different relapse groups. As shown in **Figure 3**, no obvious difference in the mRNA levels of eight genes was found among non-relapse, non-frequent relapse, and frequent-relapse groups, except for ITGB2(P = 0.006). The mRNA level of ITGB2 was significantly lower in the non-relapse group than in the non-frequent relapse (P = 0.012) and frequent relapse groups (P = 0.030).

**Figure 4:**
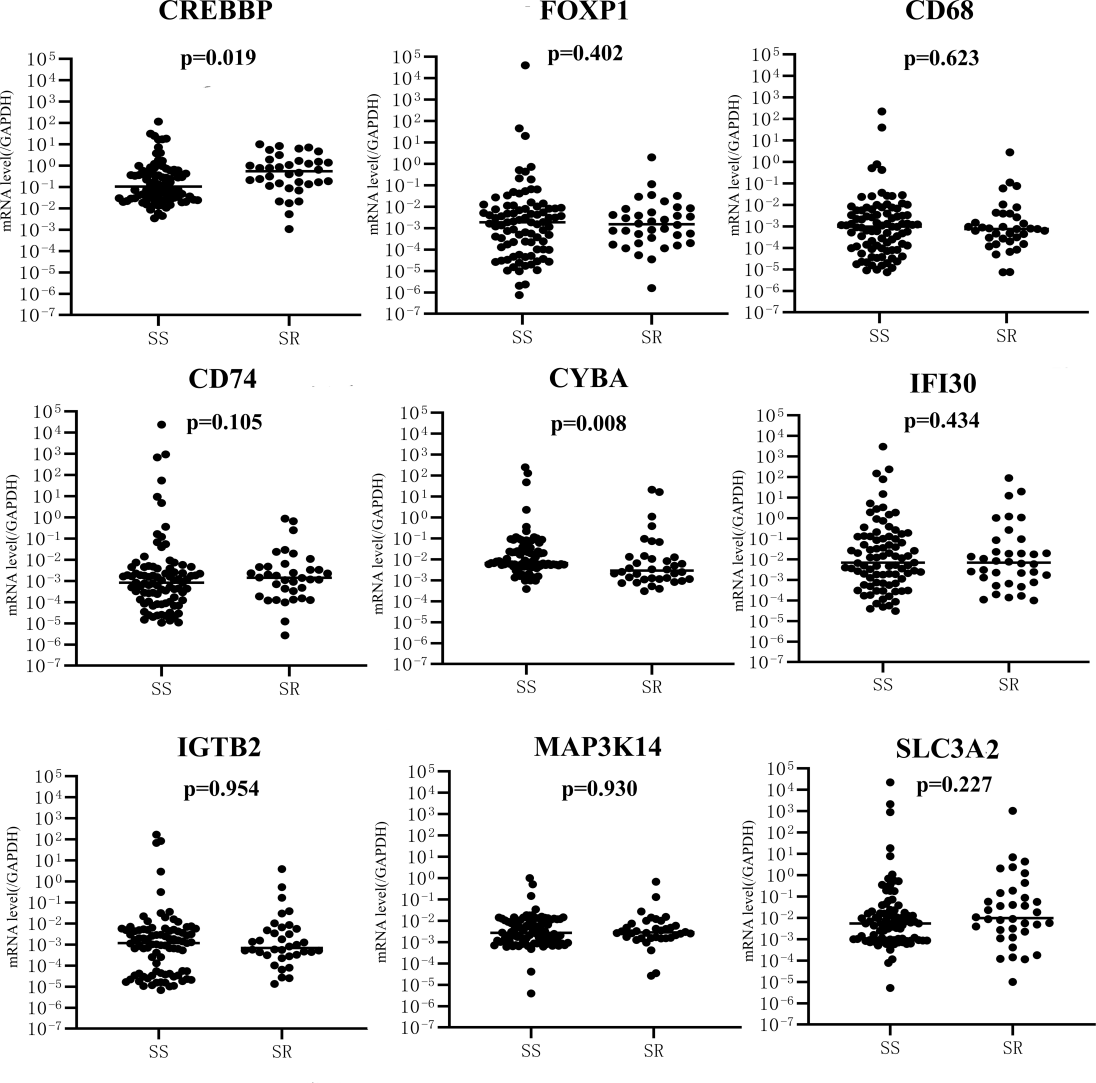
mRNA expression of nine genes between SS (*n* = 94) and SR (*n* = 35) groups. SS, SSNS; SR, SRNS.

**Figure 5:**
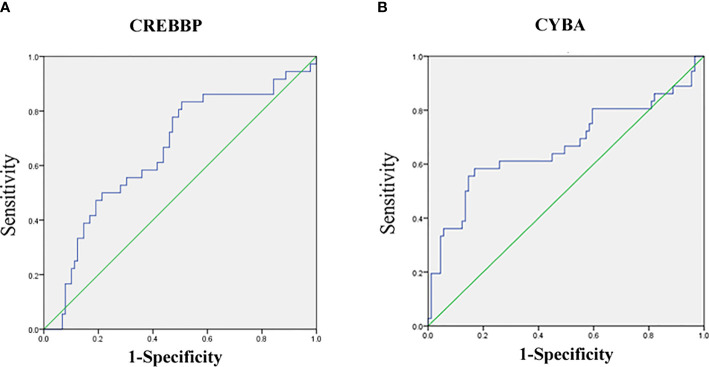
Receiver-operating characteristic (ROC) curves for distinguishing SR and SS using urinary the sediment mRNA level of CREBBP or CYBA. **(A)** ROC of CREBBP; **(B)** ROC of CYBA.

**Figure 6:**
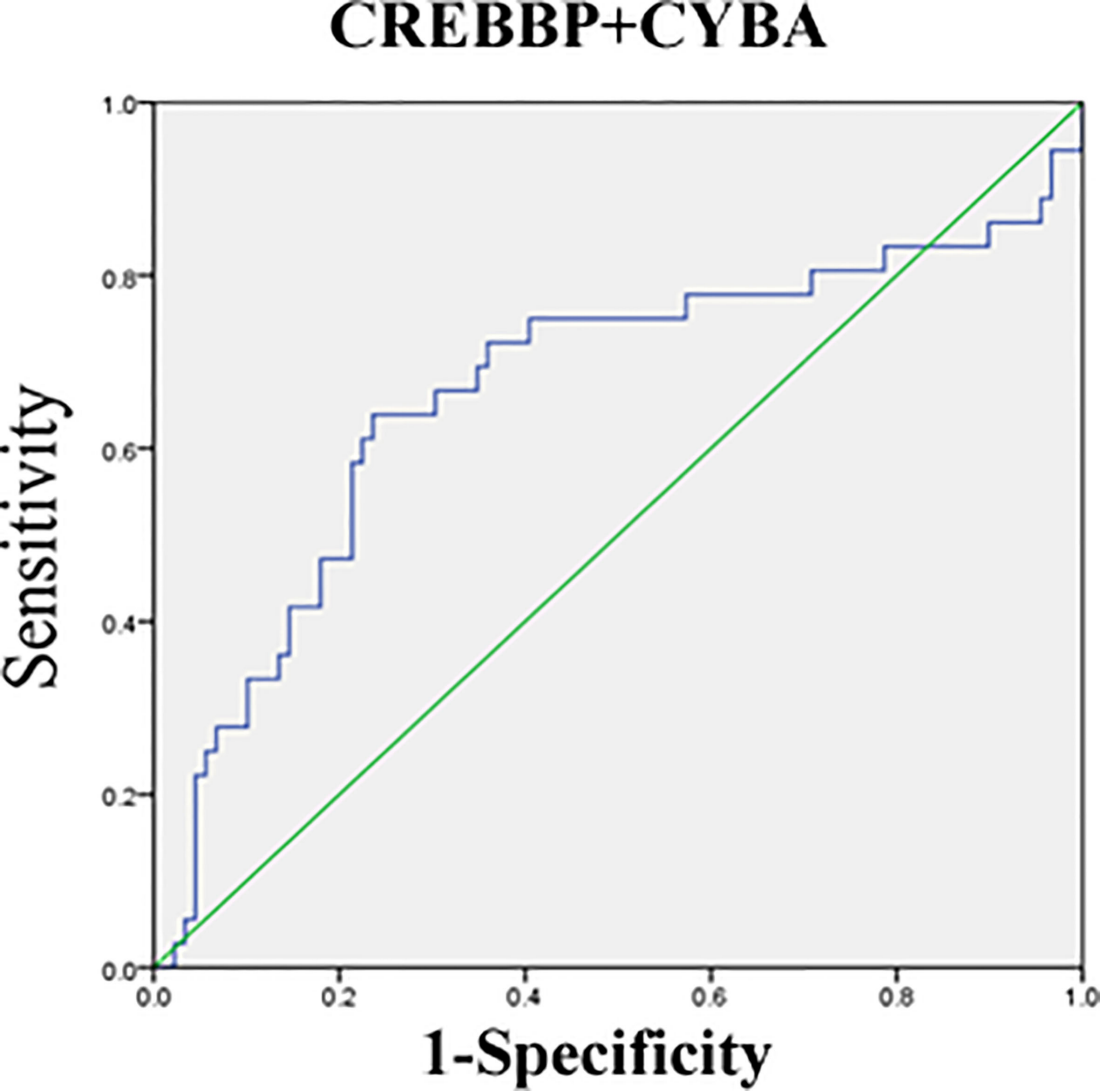
Receiver-operating characteristic (ROC) curves for distinguishing SR and SS using the combination of CREBBP and CYBA.

The mRNA level of nine genes in patients with SSNS and SRNS are shown in **Figure 4** to confirm the relationship between urinary sediment mRNA and standard steroid therapy results. CREBBP was significantly elevated in the SRNS group compared with the SSNS group (*P* = 0.02). However, in the SSNS group, CYBA significantly decreased when patients suffered from steroid resistance (*P* = 0.01) compared with the SRNS group. The effect of steroid therapy was different on CREBBP and CYBA. We evaluated the abilities of the two indicators to rule out the possibilities of SSNS and SRNS by ROC analysis. As shown in **Figure 5**, the AUCs for CREBBP and CYBA were 0.655 [95% confidence interval (CI), 0.547–0.762)] and 0.669 (95% CI, 0.549–0.788). CREBBP had a sensitivity of 83.3% and a specificity of 49.4% to rule out SSNS and SRNS (*P* = 0.007), and CYBA had a sensitivity of 58.3% and specificity of 83.1% to rule out SSNS and SRNS (*P* = 0.003). As shown in **Figure 6**, the diagnosis ability for CREBBP + CYBA was better than CREBBP or CYBA alone (AUC = 0.666; 95% CI, 0.548–0.784; sensitivity = 63.9%; specificity = 76.4%), indicating that the combination of CREBBP and CYBA was a more effective biomarker in the prediction of steroid resistance.

## Discussion

In a urinary sample, sediment cells, proteins, miRNAs, and mRNAs can be used as biomarkers to diagnose kidney injury (22–24). The number of cells in urinary sediment samples is small, and the level of total RNA is too low to be used for RNA-seq assay. Hence, in this study NanoString nCounter platform was used for the high-throughput detection of the mRNA level in urinary sediment cells from children with INS and other kidney injury diseases. The NanoString nCounter assay could detect the mRNA at a low concentration, and the lowest concentration was 20 ng/µL (21, 25). In this study, the results of the 770-gene panel revealed that 70 genes were significantly different between children with SSNS and SRNS. These differentially expressed genes were involved in pathways implicated in immune response, including Th1 differentiation, mTOR and T-cell reporter signaling, T-cell checkpoint signaling, and type II interferon signaling pathways.

The urinary sediment samples of only three patients with SSNS and three patients with SRNS were used for the high-throughput NanoString nCounter assay. The qRT-PCR verification was carried out with an expanded sample size. The levels of CREBBP and FOXP1 were found to be significantly different between INS and IgAN. FOXP1 is a member of the FOX family of transcription factors, which has a broader range of functions. Foxp1 is widely expressed and has been shown to have a role in cardiac, lung, and lymphocyte development (26). Recently, several studies have shown that FOXP1 played an important role in regulating cell development and function of the immune system, including the regulation of B-cell maturation and macrophage differentiation from monocytes. In addition, FOXP1 has a great impact on the occurrence and development of some immune diseases, such as atherosclerosis (27). FOXP1 is involved in the progression of diabetic nephropathy (28), and FOXP1 mutation is correlated with congenital anomalies of the kidney and urinary tract (29). The findings of this study indicated that FOXP1 might be a risk factor for the incidence of INS.

We found that the level of *ITGB2* mRNA in the relapse group was significantly higher than that in the non-relapse group, suggesting that the increase of *ITGB2* gene mRNA level may be a marker of INS relapse. ITGB2 (Integrin β2) is an member of integrin βfamily, encoding protein also known as CD18 (30). Its main function is to participate in cell adhesion and cell surface mediated signal transduction. It plays an important role in the recruitment and activation of polymorphonuclear neutrophils in the process of inflammation (31). Infection is one of the main causes of INS relapse in children, and INS relapse is also a process of glomerular inflammatory response. It may be the mechanism that ITGB2 involve in INS relapse in children.

When SSNS and SRNS groups were compared, CREBBP was found to be significantly elevated and CYBA was significantly lowered in the SRNS group. The CREBBP or CBP and its paralogue P300 belong to the type 3 family of lysine acetyl transferases (KAT3) of proteins known to modify histones, as well as nonhistone proteins, thereby regulating chromatin accessibility and transcription (32). CREBBP and P300 genes may be involved in a variety of cellular activities, such as DNA repair, cell growth, differentiation, and apoptosis, as well as acting as transcriptional co-activators of tumor inhibition in different signaling pathways (33). Previous studies on relapsed acute lymphoblastic leukemia (ALL) in pediatric patients identified relapse-specific mutations in CREBBP causing resistance to various drug classes (34, 35). CREBBP deficiency in mice resulted in increased effects of hormones such as adiponectin and leptin (36). Previous studies reported that the leptin level was related to the treatment of INS and adiponectin was related to SRNS at disease presentation and steroid-responsive NS relapse (37, 38). It may explain the involvement of CREBBP in drug resistance and the relapse process of INS. P22 (phox) is a ubiquitous protein encoded by the CYBA gene located on the long arm of chromosome 16 at position 24, containing six exons and spanning 8.5 kb. P22 (phox) is a critical component of the superoxide-generating nicotinamide adenine dinucleotide phosphate (NADPH) oxidases (NOXs) (39). The increase in p22phox expression can activate the production of NOXs, resulting in the increase in reactive oxygen species and then activate nuclear factor kB. It can induce oxidative stress damage (40). Hung et al. reported that the overexpression of p22phox sequestered cisplatin and caused defective cisplatin entry into the nucleus in oral squamous cell carcinoma (41). CYBA mutation or p22phox expression was closely related to insulin resistance in metabolic syndrome by affecting oxidative stress (42, 43). It indicated CYBA might affect steroid therapy in children with INS by regulating the oxidative stress pathway. We also tried to evaluate the abilities of the two indicators to exclude the possibility of SSNS and SRNS by ROC analysis. The AUC for CREBBP and CYBA was 0.655 and 0.669, respectively, indicating that CREBBP and CYBA were effective biomarkers to rule out SSNS and SRNS. CREBBP showed higher sensitivity (83.3%) but lower specificity (49.4%), while CYBA showed higher specificity (83.1%) but lower sensitivity (54.9%). This study also showed that a combination of CREBBP and CYBA was a more effective biomarker in predicting steroid resistance (AUC = 0.666; 95% CI, 0.548–0.784; sensitivity = 63.9%; specificity = 76.4%). This study also has some limitations, such as sample size of Nanostring nCounter assay is not enough and failure to verify more positive genes by qRT-PCR.

In conclusion, this study was novel in investigating urinary sediment mRNA in children with INS using high-throughput NanoString nCounter technology, and found that 70 genes may be related to SRNS. The urinary sediment mRNA level of FOXP1 was significantly lower in the non-relapse group than in the non-frequent relapse and frequent-relapse groups. Compared with the SSNS and SRNS groups, CREBBP was significantly elevated and CYBA was significantly lowered in the SRNS group. Further studies should enroll more samples and screen more genes correlated with INS and try to apply them in diagnostic methods.

## Data Availability Statement

The datasets presented in this study can be found in online repositories. The names of the repository/repositories and accession number(s) can be found below: https://www.ncbi.nlm.nih.gov/geo/query/acc.cgi?acc=GSE189734.

## Ethics Statement 

The studies involving human participants were reviewed and approved by The ethics committee of the children’s hospital of Zhejiang University, School of Medicine. Written informed consent to participate in this study was provided by the participants’ legal guardian/next of kin. Written informed consent was obtained from the individual(s), and minor(s)’ legal guardian/next of kin, for the publication of any potentially identifiable images or data included in this article.

## Author Contributions

WL and JM designed the study. WL, WX, LH, LL, and HF performed the study. WL, XS, WX, and LH analyzed the data. WL wrote the paper. WL, XS, WX, and JM revised the manuscript. All authors contributed to the article and approved the submitted version.

## Funding

This study was funded by the science and technology projects in Zhejiang Province (LGC21H200004), Key Research and Development Plan of Zhejiang Province (2019C03028) and the Medical Scientifc Projects from Health Department of Zhejiang Province (2018KY455).

## Conflict of Interest

The authors declare that the research was conducted in the absence of any commercial or financial relationships that could be construed as a potential conflict of interest.

## Publisher’s Note

All claims expressed in this article are solely those of the authors and do not necessarily represent those of their affiliated organizations, or those of the publisher, the editors and the reviewers. Any product that may be evaluated in this article, or claim that may be made by its manufacturer, is not guaranteed or endorsed by the publisher.
